# Characterizing Complex Polysera Produced by Antigen-Specific Immunization through the Use of Affinity-Selected Mimotopes

**DOI:** 10.1371/journal.pone.0005309

**Published:** 2009-04-23

**Authors:** Galina Denisova, Dimitri Denisov, Carole Evelegh, Michaela Weissgram, Jochen Beck, Stephen Ronan Foley, Jonathan Lorne Bramson

**Affiliations:** Department of Pathology and Molecular Medicine, McMaster University, Hamilton, Ontario, Canada; New York University School of Medicine, United States of America

## Abstract

**Background:**

Antigen-based (as opposed to whole organism) vaccines are actively being pursued for numerous indications. Even though different formulations may produce similar levels of total antigen-specific antibody, the composition of the antibody response can be quite distinct resulting in different levels of therapeutic activity.

**Methodology/Principal Findings:**

Using plasmid-based immunization against the proto-oncogene HER-2 as a model, we have demonstrated that affinity-selected epitope mimetics (mimotopes) can provide a defined signature of a polyclonal antibody response. Further, using novel computer algorithms that we have developed, these mimotopes can be used to predict epitope targets.

**Conclusions/Significance:**

By combining our novel strategy with existing methods of epitope prediction based on physical properties of an individual protein, we believe that this method offers a robust method for characterizing the breadth of epitope-specificity within a specific polyserum. This strategy is useful as a tool for monitoring immunity following vaccination and can also be used to define relevant epitopes for the creation of novel vaccines.

## Introduction

Proper characterization of the antibody response produced by vaccination is dependent upon the ability to characterize the specific target epitopes that are recognized by the polyserum. As an example, the HER-2 protein is over-expressed in ∼30% of breast cancer patients and is associated with poor prognosis [1], [2] making HER-2 an attractive target for the development of antigen-specific cancer vaccines. Caution is needed, however, as only antibodies to specific epitopes can suppress tumor growth [2], [3] while others actually stimulate growth [4]. As such, measurement of total levels of antigen-specific antibody is not an appropriate measure of vaccine activity since not all antibodies would be able to provide therapeutic effect. Rather, it is necessary to employ assays that permit quantification of antibodies to specific functional epitopes to adequately monitor B cell immunity.

Given the broad range of potential epitopes within a protein, characterizing the breadth of epitopes recognized by a particular polyclonal response is quite challenging. While it may be possible to monitor antibodies directed against linear epitopes using synthetic peptides, the majority of antibody responses [5] are directed at structural epitopes which are difficult to recapitulate with synthetic peptides. We have been investigating methods that employ affinity-selection of random peptides to provide an unbiased approach to epitope characterization. We have previously demonstrated that this method can be used to characterize the target epitopes of monoclonal antibodies [6], [7], [8], [9], [10], [11], [12], [13], [14] and, more recently, we have also applied this technology to polyclonal serum [15]. Manipulation and identification of random peptides is facilitated by the use of phage-display [16] where the random peptides presented on the phage surface serve as peptidomimetics of conformational and discontinuous antibody epitopes [17], [18] referred to commonly as “mimotopes”. Isolation of specific mimotopes is accomplished through affinity selection where antibodies of interest are first bound to a solid matrix and subsequently adsorbed to the phage library expressing the mimotopes. Iterative washing and adsorption steps allows for enrichment of phage carrying mimotopes which are specific for the bound antibodies. The mimotope sequences are then analyzed and assigned a location on the target protein using algorithms that we have designed [9], [15].

In the current manuscript, we have applied this method to characterize the specificity of polyclonal responses produced following immunization against a specific antigen. We have chosen HER-2 as a model antigen. As stated previously, this protein is an interesting target for vaccine development but the diverse biological effects of individual HER-2 antibodies (either stimulatory or inhibitory) necessitate careful analysis of the polyclonal repertoire following immunization. Epitope selection by antigen specific B cells will be influenced by the antigen structure within the vaccine inoculum. As such, the diversity of the polyclonal antibody response is a direct reflection of the specific vaccine formulation. To determine the utility of our mimotope technology for characterizing the epitope diversity of different polysera directed against the same protein, we examined the binding properties and epitope specificity of mouse polyserum against HER-2 generated by different vaccine inocula.

## Results and Discussion

### The composition of polyclonal responses generated in response to rat HER-2 is dependent upon the method of immunization and structure of the antigen

Mice were immunized against rat HER-2 using different strategies to determine how the method of exposure influences the antibody response. We chose to employ a xenoantigen, rat HER-2, rather than the murine homolog because our previous experience has determined that xenoantigens are more immunogenic than native antigens and yield cross-reactive immune effectors that recognize both the native and xenoantigen [19], [20], [21]. We opted to employ plasmid vaccines expressing different forms of HER-2 since this method has been shown to be highly effective in mice [22], [23], [24]. Previous work has found that similar titers of antibodies against the HER-2 extracellular domain (ECD) were evoked by immunization with plasmids expressing either the soluble ECD or full length HER-2 [24]. Given the likelihood that soluble ECD and membrane-bound ECD would adopt distinct structures and give rise to a different spectrum of antibodies, we sought to compare the properties of polysera produced by plasmid immunization with the 2 forms of HER-2. To this end, mice were immunized with plasmids expressing either the full length protein (HER-2_FL_) or the soluble extracellular domain only (HER-2_ECD_). We also observed that mice inoculated with TUBO tumors, which express rat HER-2, developed high titer antibodies against rat HER-2 (data not shown). Since the structure of the full-length protein on tumor cells may also be different than full-length protein expressed following plasmid vaccination, we included this polyserum in our analysis, as well (HER-2_TUBO_).

**Figure 1:**
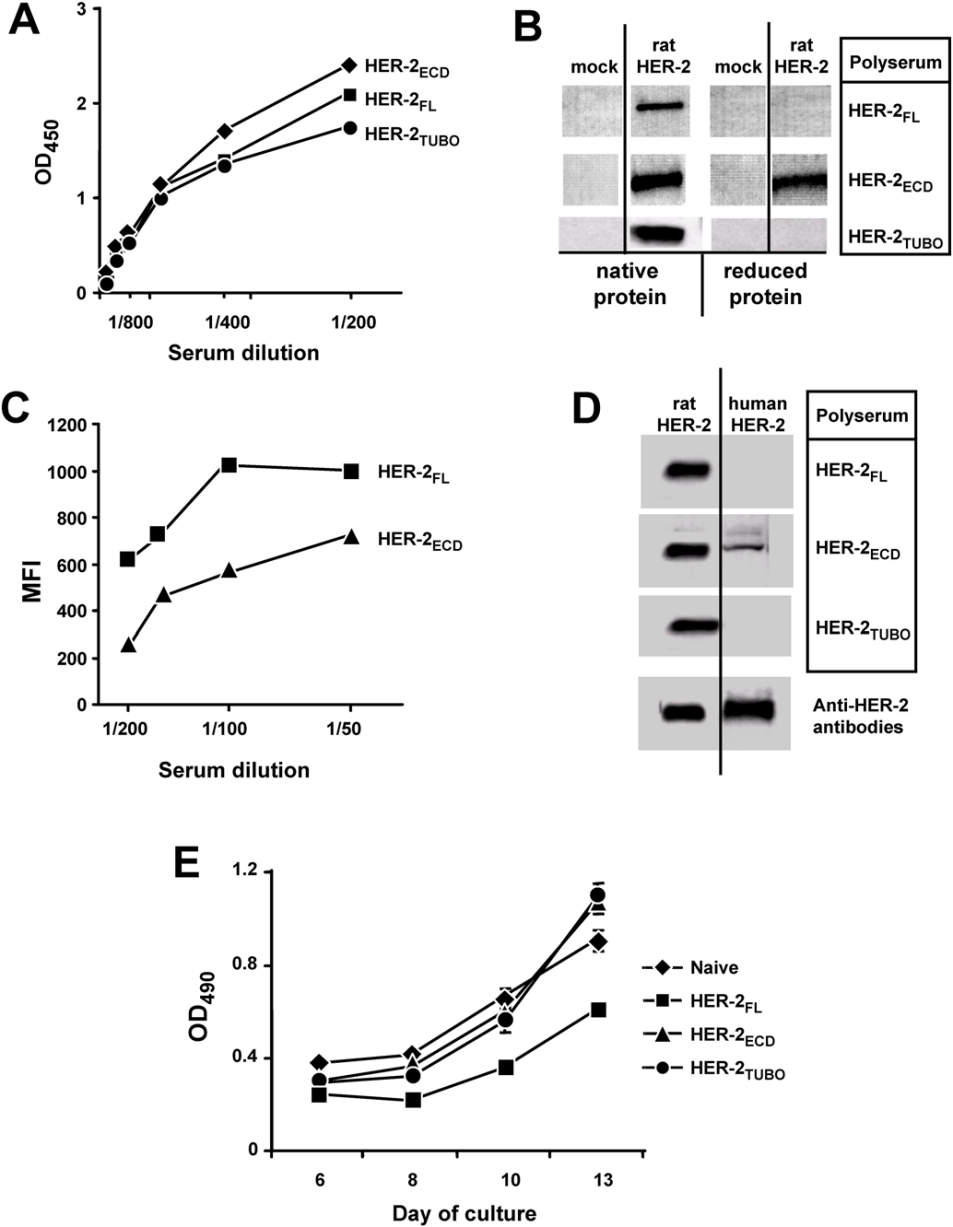
Antigen structure influences the composition of the polyclonal response. *A.* ELISA plate wells were coated with recombinant rat ECD and reacted with serial dilutions of mouse sera. *B.* Recombinant extracellular domain (ECD) of rat HER-2 was treated (*reduced protein*) or not (*native protein*) with β-mercaptoethanol and run in 10% SDS PAGE, transferred onto nitrocellulose membrane and probed with mouse sera diluted 1∶1000. *C.* 10^6^ Tubo cells over-expressing rat HER-2 were reacted with serial dilutions of mouse sera, probed with anti-mouse antibody conjugated with PE and fluorescence intensity was measured by flow cytometry. Each point is reflective of at least 10 000 events. *D.* Recombinant extracellular domain of rat HER-2 or human HER-2 were run in 10% SDS PAGE, transferred onto nitrocellulose membrane and probed with mouse sera diluted 1∶1000 or anti-HER-2 antibody. *E.* 500 TUBO cells were plated into 96 well plate in DMEM medium supplemented with 5% FBS and incubated 1–2 weeks in the presence of IgGs purified from mouse sera at a final concentration of 25 µg/ml.. The cell proliferation was measured according to manufacturer instruction by CellTiter 96® Aqueous Non-Radioactive Cell Proliferation Assay. The error bars reflect the mean+/−sem for 4 samples. These data represent the results of pooled serum from 5 vaccinated mice per group. Each experiment was replicated at least 3 times and representative results are shown.

All 3 methods yielded similar titers of antibody as determined by ELISA (Fig. 1A). Using Western blot analysis, we observed that the polyserum generated in response to HER-2_ECD_ recognized both native and denatured forms of HER-2 equally well (Figure 1B). By contrast, binding of HER-2 by HER-2_FL_ and HER-2_TUBO_ polyserum was completely ablated by denaturation (Figure 1B). Since it appeared that the antibodies generated in mice exposed to full-length HER-2, either through plasmid vaccination or exposure to tumor cells, have distinct specificity relative to the antibodies produced in response to HER-2_ECD_, we examined the ability of the sera to recognize native HER-2 expressed on the surface of TUBO cells. For this experiment, TUBO cells were coated with various dilutions of serum and the quantity of bound antibody was determined by flow cytometry. Unfortunately, we could not employ this method to study polyserum produced by TUBO cells themselves because we could not distinguish HER-2 specific antibody from other TUBO-specific antibodies. Nonetheless, these data demonstrate that although the HER-2_ECD_ and HER-2_FL_ polysera exhibit comparable titers of anti-HER-2 antibody by ELISA (Figure 1A), the HER-2_FL_ polyserum had markedly enhanced capacity to bind the native protein (Figure 1C), suggesting that the polyserum produced by HER-2_FL_ may be more specific for the native form of the protein. Given the observation that the HER-2_ECD_ polyserum was less sensitive to protein structure, we hypothesized that this polyserum would also prove to be more cross-reactive. Indeed, when the various polysera were tested for cross-reactivity by Western blot, to another form of HER-2 (human HER-2), the HER-2_ECD_ polyserum revealed some cross-reactivity whereas the HER-2_FL_ and the HER-2_TUBO_ showed no binding to the human protein (Figure 1 D). Thus, although all 3 polyserum exhibit similar titers of anti-HER-2 antibodies by ELISA using native protein, it is clear that each polyserum is composed of a discrete combination of individual antibody specificities.

### Differential polyserum compostion correlates with impact on tumor growth in vitro

To gain further insight into possible differences in the polysera, we tested whether the anti-HER-2 antibodies would suppress TUBO cell proliferation *in vitro*. TUBO cells were grown for 2 weeks in the presence of IgG purified from the various polysera (25 µg/ml final). Only IgG purified from the HER-2_FL_ serum suppressed tumor cell growth whereas the other sera had no effect (Figure 1E; p<0.01 based on ANOVA and post-*hoc* Tukey test). These results again demonstrate that the polyserum produced by the 3 immunization methods have different functional properties. Notably, even though both sera produced in response to full-length protein (HER-2_FL_ and HER-2_TUBO_) display similar dependence upon protein structure for binding, the functional effects of these polysera appear to be distinct. This difference may reflect differential abundance of inhibitory and activating antibodies.

### Characterization of eptiope specificity within the anti-HER-2 polysera using affinity-selected random peptides

Characterization of epitope specificity for antibodies can be quite challenging, particularly in the case of structural epitopes. Since binding of both HER-2_FL_ and HER-2_TUBO_ sera to recombinant HER-2 is abrogated when the protein is denatured, it would appear that the epitopes recognized by these sera are largely conformational. Therefore, to characterize the epitope-specificity of these sera, we opted to employ a mimotope-based strategy where random circularized peptides displayed on recombinant phage are affinity-selected using specific polyserum and a computer algorithm is employed for epitope prediction based on the sequence of the selected mimotopes [9], [15]. To facilitate the manipulation and identification of these random peptides, we have employed a phage-display method where random 10-mer circularized peptides are expressed on N-terminus of the phage major coat protein. Since unrelated antibodies present in mouse serum can confound our analysis, the phage library was adsorbed to naïve mouse serum prior to further analysis to remove all phages that were reactive with general mouse antibodies. Likewise, anti-phage antibodies present in mouse serum would also complicate interpretation, so the sera to be tested was adsorbed to immobilized wild type phage fd-tet [25] to remove phage-specific antibodies. Phage bearing specific mimotopes were enriched through iterative immunoaffinity selection steps on immobilized anti-mouse IgG. Specific phages were defined as those which bound to serum from immunized mice but not to serum from naïve mice. The sequences of the specific mimotopes selected through iterative rounds of screening are presented in Table 1. Interestingly, we did not find any common mimotopes in the collection of sequences selected by each individual polyserum suggesting that each serum is composed of distinct antibodies. It is also notable that we only uncovered a limited set of phages with HER-2_TUBO_ serum, suggesting that this polyserum has a limited range of specificity.

**Table 1:** Mimotope sequences of affinity-selected phage.

HER-2_ECD_ a mimotopes		HER-2_FL_ mimotopes		HER-2_TUBO_ mimotopes	
Phage	Mimotope sequence	Phage	Mimotope sequence	Phage	Mimotope sequence
ECD.1	KAEERFHEVR	FL.1	NWSIAGD	TUBO.1	TYSGRAACGV
ECD.2	WNIFQSLSSP	FL.2	SRGDTEP	TUBO.2	SASMPFPANE
ECD.3	RVAATAAREE	FL.3	TSDSWQR	TUBO.3	SKPKYEKPSQ
ECD.4	TPEPLEEAAS	FL.4	PKTEVPQ	TUBO.4	EVTTEHVEAN
ECD.5	DHVFKRPQPS	FL.5	DDFSPPR	TUBO.5	RSHERHPPSP
ECD.6	ETCVEKNEAD	FL.6	EWYTPQG	TUBO.6	DDELHSGTSY
ECD.7	WRRDGC	FL.7	SRCDLDEKGC	TUBO.7	SPPAFSHPMQ
ECD.8	YMDPHTQREA	FL.8	PRCKHNQKKC	TUBO.8	VNTQGPNSIA
ECD.9	EPVRDNCAPS	FL.9	AGACKRMREF		
ECD.10	VERDIATRPW	FL.10	ATPKRTHDHD		
ECD.11	ERPEIEDVCQ	FL.11	TSNNRVEARA		
ECD.12	QKTLNTSNAN	FL.12	RTTPSGGGFK		
ECD.13	VKTLNTSNAN	FL.13	DGWYVAQ		
ECD.14	RDTTMWEVNA	FL.14	GGRWGES		
ECD.15	WGHCSQGMIE	FL.15	WYTTPGS		
ECD.16	PPFDVFHNPM	FL.16	SGWYTPV		
ECD.17	DTHAGMHSPT	FL.17	AKDEQPM		
ECD.18	RMPHHDPQLM	FL.18	GWYVSSP		
ECD.19	TQNLMQMQHA	FL.19	PSEGQSE		
ECD.20	SPFMLMHGEH	FL.20	GTAGGEITEH		
ECD.21	TCQAGRESMHNP	FL.21	FYSSMFWAVGEQ		
ECD.22	WETMHNPGTP	FL.22	LQEFPGDQLV		
ECD.23	DALNMHEGRP	FL.23	LSTRLWIPAW		
ECD.24	SKLETTMHSP	FL.24	KTTTWPSTPT		
ECD.25	LGGMDSMHSP	FL.25	MWASVNKMA		
ECD.26	NTAHSDMHSP	FL.26	PPPLLAGDPK		
ECD.27	MAQMHEPVRS	FL.27	AVNQCTTVLA		
ECD.28	NTAHSDMHSP	FL.28	GTAGGEITEHE		
ECD.29	LPQHNMMHDP				
ECD.30	PSNYSAMHAP				
ECD.31	RCETHFNMHEPY				

a Polyserum used for mimotope isolation.

**Figure 2:**
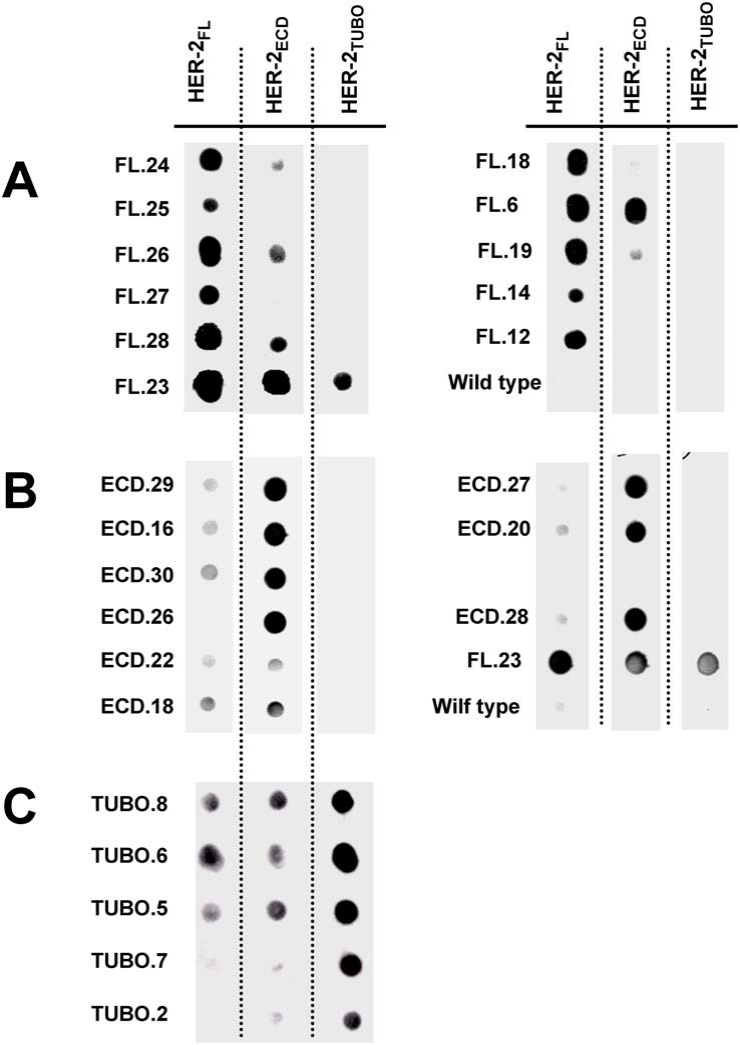
Limited cross-reactivity between phage selected with different polysera. Selected phage were immobilized onto nitrocellulose membranes and reacted with three different sera overnight at 4°C (HER-2_FL_ – serum prepared from mice immunized with full-length HER-2; HER-2_ECD_ – serum prepared from mice immunized with soluble protein; HER-2_TUBO_ – serum from tumor bearing mice). Bound antibodies were detected with goat anti-mouse IgG HRP conjugated antibody and the signals were developed by ECL. *A.* phages selected with HER-2_FL_serum, *B.* phages selected with HER-2_ECD_-serum, *C.* phages selected with HER-2_TUBO_ -serum).

To further examine the “specificity” of the affinity-selected phages, representative phages from each collection were hybridized with each of the 3 polysera using dot blot. In each case, the selected phage hybridized best with the sera used for the affinity purification (Figure 2). Even though we did not isolate mimotopes using HER-2_FL_ serum that matched the sequence of the mimotopes selected with HER-2_ECD_ serum, we did find 2 phage isolated with HER-2_FL_ that could be recognized by both sera (FL.6 and FL.23) (Figure 2A). Notably, phage FL.23 was bound by all 3 sera. In the case of the phage isolated with HER-2_ECD_ serum, although we observed low-level cross-reactivity with HER-2_FL_ serum, we observed no cross-reactivity with HER-2_TUBO_ serum (Figure 2B). With regard to the phage selected by HER-2_TUBO_ serum, comparable, low-level cross-reactivity was seen with both HER-2_FL_ and HER-2_ECD_ (Figure 2C).

Given the limited cross-reactivity between the affinity-selected phages, it appeared as though the selected phage may reflect a molecular “signature” of the polyclonal specificity. One way to describe such a “signature” would be the analysis of the specific amino acid residues within each collection of phages. Each collection of phages was dominated by a distinct set of amino acid residues (Table 2) resulting in a distinct hierarchy of amino acid frequency with the various collections. Furthermore, when we examined the frequency of specific amino acid pairs, which reflects the minimal homology elements that are employed by the computer algorithm for epitope prediction [9], [11], we found that the majority of generalized pairs were unique to each collection (Table 3, bold letters) and only a few pairs were found in more than 1 mimotope collection (Table 3, italicized). These results demonstrate that the phage selected through our affinity-purification method represent a “signature” of the respective polyserum used for purification.

**Table 2:** Occurrence of statistically significant amino acids within mimotope collections.

HER-2_ECD_ a mimotopes		HER-2_FL_ mimotopes		HER-2_TUBO_ mimotopes	
Residue	Frequency^b^	Residue	Frequency	Residue	Frequency
**Glu**	10.07%	**Gly**	10.29%	**Ser**	15.00%
Pro	9.06%	Thr	9.88%	Pro	13.75%
**Ser**	8.05%	Pro	9.05%	**Glu**	8.75%
Ala	7.72%	**Ser**	8.23%	Ala	8.75%
Arg	7.38%	Ala	7.00%	His	6.25%
Thr	7.05%	**Glu**	7.00%	Thr	6.25%
His	6.38%	Arg	5.35%	**Gly**	5.00%
Asn	6.38%	Asp	5.35%	Asn	5.00%
**Met**	5.70%	Trp	5.35%	Val	5.00%
Asp	4.36%	Lys	4.94%	Arg	3.75%
**Gly**	4.03%	Gln	4.53%	Tyr	3.75%
Cys	3.36%	Val	4.12%	Gln	3.75%
Val	3.69%	Leu	3.29%	Lys	3.75%
Gln	3.69%	Asn	2.47%	Phe	2.50%
Leu	3.02%	Cys	2.47%	Met	2.50%
Trp	3.02%	Phe	2.47%	Asp	2.50%
Lys	2.01%	Tyr	2.47%	Cys	1.25%
Tyr	2.01%	His	2.06%	Ile	1.25%
Phe	1.68%	Met	2.06%	Leu	1.25%
Ile	1.34%	Ile	1.65%		

a Polyserum used for mimotope isolation.

b The frequency was determined as: (# specific residues/total # of residues in the collection) ×100.

**Table 3:** Occurrence of statistically significant amino acid pairs within mimotope collections.

HER-2_ECD_ a mimotopes		HER-2_FL_ mimotopes		HER-2_TUBO_ mimotopes	
Generalized Amino Acid Pairs^b^	Occurrence within mimotope collection^c^	Generalized Amino Acid Pairs	Occurrence within mimotope collection	Generalized Amino Acid Pairs	Occurrence within mimotope collection
**MH** d	11	**OO**	10	**OY**	2
**JO**	8	*OP*	9	**PA**	2
*OP*	8	**GJ**	7	*AX*	2
**BJ**	7	**OJ**	7	**OH**	2
**HO**	7	**AG**	5	*BP*	2
**JP**	6	*ZY*	5	**HP**	2
**BP**	5	**GG**	5	*PP*	2
**HJ**	5	**PB**	5	**MX**	1
**OM**	5	**GZ**	4	*PM*	1
*AX*	4	**JZ**	4	**MP**	1
**XM**	4	*PP*	4		
**XA**	4	**JH**	3		
**JA**	4	**XX**	3		
**GM**	2	*PM*	2		
**HX**	2				
*ZY*	2				
**AH**	2				

a Polyserum used for mimotope isolation.

b Single letters amino acid codes (See Methods).

c Number of mimotopes carrying this generalized pair within the specific collection.

d Unique pairs are presented in bold, pairs shared by two datasets are presented in italics.

### Affinity-selected phage can be used to predict epitopes targets of the polyserum

**Figure 3:**
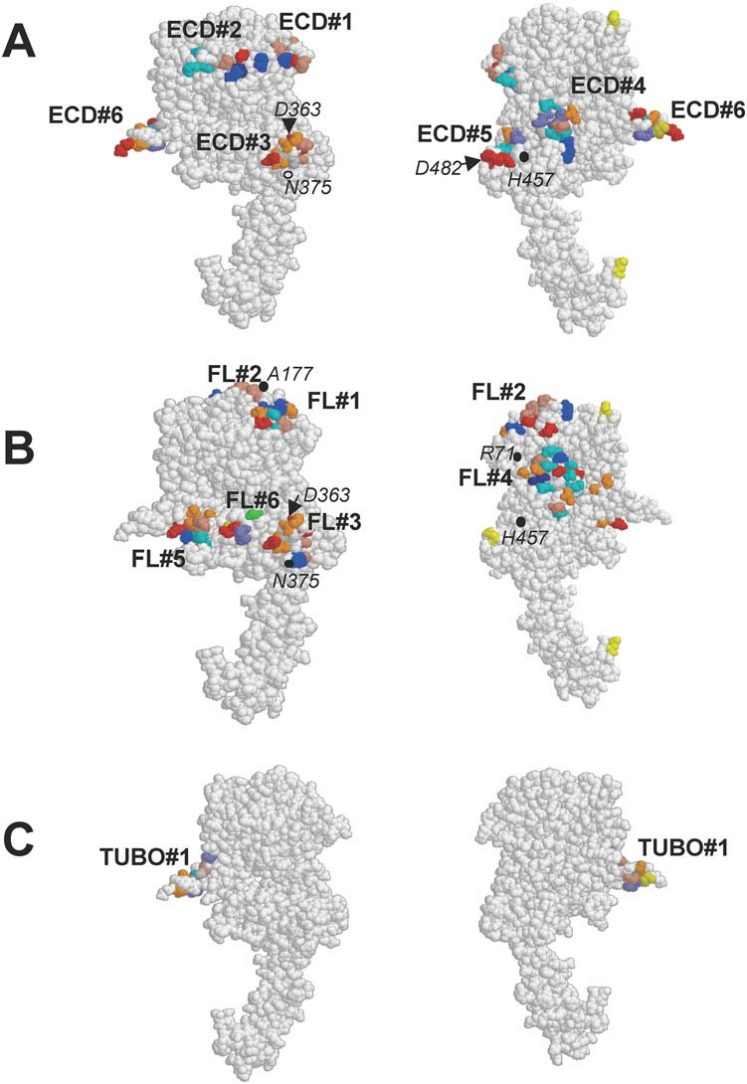
Three-dimensional modeling of putative epitopes. Three-dimensional model of rat HER-2 is presented in two orientations. *A.* clusters found for HER-2_FL_serum, *B.* clusters found for HER-2_ECD_-serum, *C.* clusters found for HER-2_TUBO_ –serum. Serum- specific clusters of amino acid pairs are numbered. Amino acid are coloured according to RasMol amino acid colour code: red = Glu and Asp, blue = Arg and Lys, brown = Pro, orange = Ser and Thr, Cyan = Asn and Gln, violet = Trp and Phe, purple = His.

Of interest, we also sought to determine whether the affinity-selected mimotopes could be used to elucidate the epitopes recognized by the polyserum. To attribute the mimotope sequences to specific epitopes on the protein, we used our novel computer algorithm [9] which categorizes mimotopes based on minimal homologies (i.e. neighbouring amino acids) into clusters defined by the position of these minimal homologies on the folded protein. These clusters represent hypothetical epitopes. As a result of cluster analysis performed using stringent conditions where we only included amino acid pairs determined to be significant based on their actual occurrence compared to the estimated random occurrence [11], a numbers of distinct clusters were found on the surface of the protein (Figure 3, Table 4). Six epitopes were predicted for the HER-2_ECD_ polyserum (ECD#1–ECD#6; Figure 3A; Table 4), six epitopes were predicted for the HER-2_FL_ serum (FL#1–FL#6; Figure 3B; Table 4) and a single epitope was predicted for HER-2_TUBO_ serum (TUBO#1).

**Table 4:** Clusters predicted by computer algorithm.

Cluster #	Amino acids	Cluster #	Amino acids	Cluster #	Amino acids
ECD#1	98–100, 113, 135, 138, 162	FL#1	137–138, 164–169	TUBO#1	246, 248–249, 252, 260–264
ECD#2	86–90, 155–157, 159–160	FL#2	172–173, 175–176, 179		
ECD#3	336–338, 359, 361, 363–366, 369–370	FL#3	336–338, 359, 361–363, 365–367, 370, 372–373		
ECD#4	1, 26, 29, 445, 466–471	FL#4	32, 35–36, 50–51, 53–57, 59, 73, 75–76, 84, 467–468		
ECD#5	474–475, 477, 480–482	FL#5	284–286, 289,291, 295–299, 311–312		
ECD#6	249, 252, 259–263, 265	FL#6	323, 326–328, 331		

**Figure 4:**
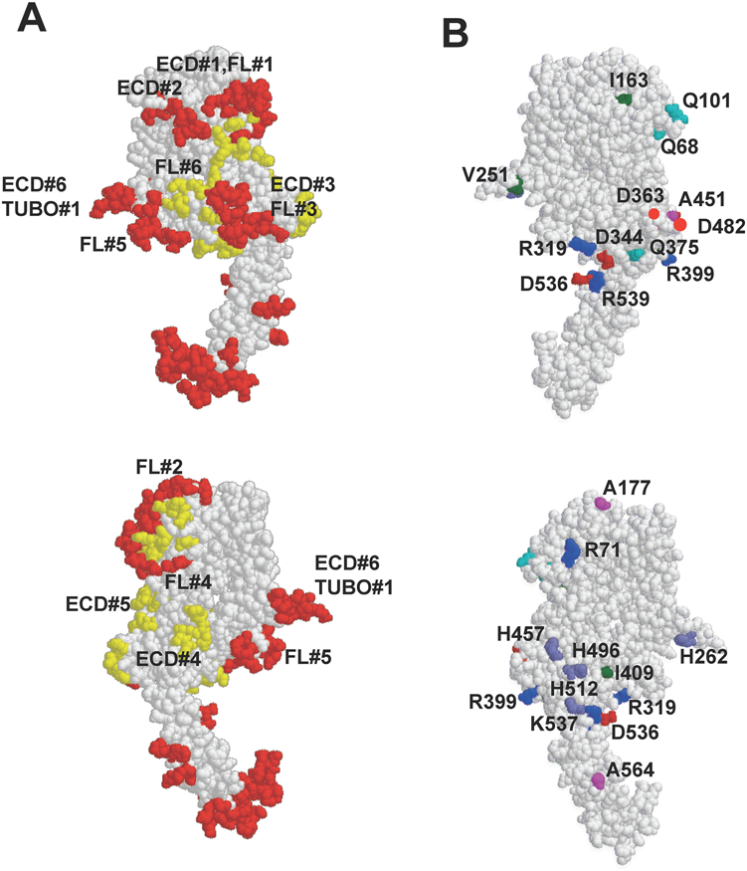
Three-dimensional modeling of predicted epitopes and non-conserved amino acids. *A.* Epitopes predicted by the DiscoTope method. Immunodominant epitopes are coloured in red, epitopes of lower immunogenicity are shown in yellow. *B.* Map reveals surface exposed amino acids on the rat HER-2 extracellular domain that differ from the corresponding residues on mouse HER-2. Amino acid are coloured according to RasMol amino acid colour code: red = Glu and Asp, blue = Arg and Lys, brown = Pro, orange = Ser and Thr, Cyan = Asn and Gln, violet = Trp and Phe, purple = His.

To determine whether the epitopes predicted by our affinity-selected mimotopes are consistent with regions predicted to be immunogenic, we employed the DiscoTope computer algorithm to predict B-cell epitopes based on protein structure [26] (Figure 4A and Table 5). Regions predicted to be highly immunogenic are shown in red and regions predicted to be moderately immunogenic are shown in yellow. Strikingly, most of the epitopes predicted by our method mapped to regions predicted to be immunogenic based on independent criteria (Figure 4A) which offers additional confidence in the epitopes predicted by our method.

**Table 5:** Theoretical prediction of HER-2 epitopes.

Amino acids	Maximal potential*	Amino acids	Maximal potential*
11–14, 17–19, 22	0.85	337–338, 344–345, 347	0.72
42, 44–48, 50, 53	1.13	357–358, 360–375	1.55
66, 68–71, 75	0.65	377–378, 380–381	0.5
87–89, 96, 98–101, 113–115, 117	1.7	395–399	0.9
135–139	0.96	462–463, 465–468	0.78
155, 157–158, 160–173, 175–177, 179, 181	1.25	475–476, 481–482, 485–487	0.64
186–189	0.42	526–530, 535–536	0.9
251–253, 259–265	1.67	549–552	1.13
295–297, 301–307	1.59	577–578, 580–601	1.74
311–319	1.12	603–608	1.3
327–328, 330–333	0.89		

* potential calculated for every amino acid using DiscoTope software and characterizing immunogenic property of a given amino acid.

The C-terminus of the HER-2 ECD was predicted to be strongly immunogenic by DiscoTope, yet none of the polyserum mapped to this site. Interestingly, this site exhibits 100% homology between mouse and rat proteins (Figure 4B). In general, the cluster analysis indicates that antibody responses are directed against epitopes on rat HER-2 where there are differences between rat and mouse sequences. Clusters ECD#1 and FL#1 are both located in a similar region of domain 1 close to N103 (this residue is a putative glycosylation site in mouse HER-2 but not in rat HER-2 due to replacement T105 in the mouse protein with A105 in the rate protein). Common clusters ECD#3 and FL#3 are located within domain 3 around replacements of D363 for N and Q375 for H in the mouse protein. Cluster FL#4 is located on domains 1 and 3 close to replacement of R71 for H and close to cluster ECD#4 (close to replacements H457 for N). Cluster ECD#5 is located at the beginning of domain IV around replacement of D482 for A in mouse protein. Cluster FL#2 is located on loop 168–182 of domain 1 close to replacement of A177 for T. Thus, similar to T cell epitopes, B cell epitopes in xenoantigens may also be generated by specific non-conserved amino acids within the vicinity of the epitope which enables the B cells to overcome tolerance.

### Conformational analysis reveals distinct epitopes within similar regions

It is interesting to note that although many of these epitopes map to similar regions of the protein, for the most part, they involve distinct amino acid residues (Table 4). We have observed similar outcomes using monoclonal antibodies directed against the West Nile virus E protein [9]. The differences in epitope selection noted in this manuscript probably reflect differences in conformation of the soluble and cell-attached proteins which alter the way the protein is bound by antigen-specific B cells. We found that most of the clusters are located at protruding loops or β-turns exposed on the protein surface as it was shown for many other epitopes [26]. Of interest, epitopes ECD#3 and FL#3 overlap and involve many, but not all, of the same residues. These 2 clusters are located at two closely positioned turns connecting two β-strands and two anti-parallel α-helices. It is also interesting that clusters ECD#6, FL#5 and TUBO#1 are all located within the area of the epitope identified for pertuzumab [27], a therapeutic antibody currently under development. The pertuzumab epitope encompasses three loops. ECD#6 and TUBO#1 include the first loop of the pertuzumab epitope while cluster FL#5 includes the two adjacent loops. Although TUBO#1 overlaps with ECD#6, these two clusters only share 3 common residues. It is also striking that the HER-2 specific antibodies that were generated in response to growing TUBO tumors seemed to identify only a single cluster which is consistent with the limited number of mimotopes isolated by affinity selection with the HER-2_TUBO_ polyserum (Table 4). It is also notable that overlapping clusters FL#4 and ECD#4 are planar surfaces formed by β-sheets and located close to each other in domain III; however FL#4 is shifted in the direction of domain I and is actually located at the interface between domain I and domain III.

Antibody targets are defined by physico-chemical and geometrical properties such as hydrophilicity [28], chain flexibility [29], [30], surface accessibility [38] and protrusion from the surface [31]. Changes of antigen conformation and its participation in various complexes should lead to corresponding changes in immune response [32], [33], [34]. Our results indicate that the method of immunization has a pronounced effect on the breadth of the antibody response against a given protein. Affinity-selected mimotopes can be used as a tool for characterizing these differences and providing a “signature” of the polyclonal response. Using predictive tools, this signature can also be used to characterize the specific binding sites for the antibodies which can offer insight into their functionality. Through iterative application of this technology in human studies of antibody-mediated resistance, it should be possible to develop “resistance” signatures which would be indicative of effective vaccination. Additionally, knowledge of mimotopes associated with specific “functional” domains may serve as the basis for developing epitope-specific vaccines.

## Methods

### Plasmid vaccines and immunizations

The extracelluar domain of rat HER was amplified from cDNA using the primers 5′GTCGAAGCTTATGGAGCTGGCGGCCTGG and 5′ GACTGAATTCTTAGTTGATGGGGCACGG and inserted into the vaccination plasmid pcDNA3.1-002 [35] to yield prHER-2_ECD_. Full length rat HER-2 was prepared by PCR amplification of full length HER-2 gene using primers 5′-GCCCGGTGACATAAATCATTGCAACTGTAG and 5′-CTACAGTTGCAATGATTTATGTCACCGGGC and inserted into pcDNA3.1-002 to yield prHER-2_FL_. Balb/c mice (6–8 weeks old) were immunized by intramuscular injection followed by electroporation [36] with 100 µg of pHER-2_ECD_ or pHER-2_FL_. One month later, the immunization was repeated. Serum antibodies were measured 2 weeks following the booster immunization. All animal work described in this manuscript was approved by the Animal Research Ethics Board of McMaster University.

### TUBO breast cancer tumor model

The TUBO cell line, kindly provided by Dr. Guido Forni (Department of Clinical and Biological Sciences, University of Turin, Orbassano, Italy), was derived from a spontaneous mammary tumor which arose in a BALB NeuT transgenic mouse expressing the rat neu oncogene [37]. TUBO cells grow progressively in normal BALB/c mice and give rise to tumors which are histologically similar to those in BALB NeuT mice. 10^6^ TUBO cells were injected subcutaneously into Balb/c mice and the recipients were monitored for tumor growth. For the experiments described in our manuscript, serum was obtained from peripheral blood isolated one month after tumor cell inoculation.

### Proliferation assay

500 TUBO cells were plated into a 96 well plate in DMEM medium supplemented with 5% FBS and incubated for 1–2 weeks in the presence of serum IgGs [purified on Protein G-Sepharose (GE Healthcare Bio-Sciences AB, Uppsala, Sweden)] diluted at final concentration of 25 µg/ml. Cell proliferation was measured using the CellTiter 96® Aqueous Non-Radioactive Cell Proliferation Assay (Promega, Madison, WI, USA) according to the manufacturer's instructions.

### Production of recombinant HER-2

Recombinant HER-2 was used for Western blot and ELISA. To provide a source of recombinant HER-2, the extracellular domains (without the transmembrane domain) of rat and human HER-2 were subcloned into pcDNA3.1-002 [35] to yield prHER-2_ECD_ and phHER-2_ECD_. The plasmids were subsequently transfected into 293T cells using Lipofectamine2000 (InVitrogen). Supernatants were collected and the samples were concentrated using a Vivaspin 50000 column (Sartorius AG, Goettingen, Germany). We also subcloned the cDNAs into pCMVhygro which encodes the hygromycin resistance gene downstream of an IRES to yield pCMVhygro-rHER-2_ECD_ and pCMVhygro-hHER-2_ECD_. These plasmids were transfected into CHO cells and stable transfectants were selected using 250 µg/ml of hygromycin (Roche Diagnostics, Mannheim, Germany) and then cloned by limited dilution. CHO cells expressing high levels of HER-2 ECD were identified by Western blot of culture supernatants. Similar to the 293T-derived HER-2 ECD, supernatants from CHO cells expressing high-levels of HER-2 ECD were concentrated by centrifugation on Vivaspin 50000 columns (Sartorius AG, Goettingen, Germany).

### Western blotting

Recombinant HER-2 proteins from the 293T cultures were mixed with gel-loading buffer. For some experiments, the proteins were also treated with β-mercaptoethanol. The proteins were electrophoresed through 10% SDS-PAGE, transferred onto nitrocellulose membrane and probed with mouse sera diluted 1∶1000. Bound antibody was detected using goat anti-mouse IgG conjugated with HRP (Jackson Immunoresearch Laboratories Inc. West Grove, Pennsylvania, USA) and the ECL chemiluminescent reagent (Amersham Biosciences, NJ, USA). As positive controls for HER-2 detection, we employed a monoclonal antibody against rat HER-2 (BD Biosceinces, catalog# 610161) and a polyclonal serum against human HER-2 (R&D Systems, catalog #AF1129)

### Measurement of HER-2 specific antibodies by ELISA

96 well plates (Nunc, New York, USA) were coated with rat HER-2 produced from the CHO cultures. Wells were blocked with 5% non-fat milk in Tris-buffered saline (TBS, pH 7.4), and reacted with serial dilutions (in TBS/0.5% non-fat milk) of mouse sera. The signal was developed with substrate Sigmafast OPD (Sigma Aldrich, St. Louis, USA) after incubation with goat anti-mouse IgG conjugated with HRP.

### Measurement of HER-2 specific antibody by FACS

10^6^ TUBO cells, which express high levels of rat HER-2, were incubated with serial dilutions of mouse sera. The TUBO cells were then washed to remove free mouse antibodies and incubated with goat anti-mouse IgG conjugated with phycoerethrin (PE) ((Jackson Immunoresearch Laboratories Inc. West Grove, Pennsylvania, USA). Fluorescence intensity was measured using an LSRII flow cytometer (BD Biosciences). MFI was calculated on a minimum of 10000 events.

### Phage display library screening

We employed a phage display library presenting random peptides with the structure XC(X)_10_CX inserted into N-terminus of pVIII, a major coat protein of filamentous bacteriophage, where X represents any amino acid (kindly provided by Dr. J. Scott, Simon Fraser University) [38]. Screening of the phage library was performed according to the method described by Smith [39]. The library was initially depleted of phages recognized by naïve mouse serum by 3 sequential pannings of the library with immobilized serum of non-immunized mice. The resultant “depleted” library was used for mimotope identification. Similarly, the sera obtained from immunized mice were initially depleted of anti- phage activity by overnight incubation at 4°C with wild type phage immobilized on plastic plates. The sera were also depleted of IgM by similar incubation with immobilized anti-IgM antibody. Serum specific peptides were selected by two rounds of iterative affinity selection and specificity of phages for different mouse sera was assessed by dot blot (according to the method described in [40]). Briefly, phages were immobilized onto nitrocellulose membranes. The membranes were then blocked with 5% non-fat milk in TBS and probed with depleted mouse sera (1∶1000 in TBS/0.5% non-fat milk). Bound antibodies were detected using goat anti-mouse IgG conjugated with HRP and the ECL chemiluminescent reagent. DNAs from the serum-specific phages were subsequently purified and the pVIII inserts were sequenced and translated to amino acids for further analysis.

### Computer algorithm

Amino acid sequences of affinity-selected phages were used as a database for the computer algorithm [9], [11] to perform a cluster analysis. The algorithm is based on the assumption that affinity-selected peptides must share key contact residues with the true epitopes in the native protein. In the three-dimensional structure of a protein, amino acids creating the epitope can be discontinuous and become juxtaposed at the surface of the antigen through protein folding. Therefore, we assume that neighboring amino acids within the selected phage-displayed peptides may represent pairs of juxtaposed amino acids residues on the protein surface. All conserved residues were consolidated into six functional subgroups of amino acids and given single-letter codes: Arg, Lys = B; Glu, Asp = J; Ser, Thr = O; Leu, Ile, Val = U; Asn, Gln = X; Trp, Phe = Z. Additionally, we assume that the most frequently observed tandem amino acid pairs within a given set of affinity-selected peptides likely represent the contact amino acids within the epitope. The computer algorithm was used to find clusters of these pairs on a surface of HER-2 protein by analyzing its three-dimensional model. Three-dimensional models of HER-2 were created by RasMol software (http://www.bernstein-plus-sons.com/software/rasmol/doc/rasmol.html).

To corroborate the predictive power of our strategy, we also employed the DiscoTope software [41] which predicts antibody epitopes based on “a combination of amino acid statistics, spatial information, and surface exposure”. It is created on the basis of a compiled data set from 76 X-ray structures of antibody/antigen protein complexes.
